# Type I IFN induces long-chain acyl-CoA synthetase 1 to generate a phosphatidic acid reservoir for lipotoxic saturated fatty acids

**DOI:** 10.1016/j.jlr.2024.100730

**Published:** 2024-12-14

**Authors:** Shelley Barnhart, Masami Shimizu-Albergine, Eyal Kedar, Vishal Kothari, Baohai Shao, Melissa Krueger, Cheng-Chieh Hsu, Jingjing Tang, Jenny E. Kanter, Farah Kramer, Danijel Djukovic, Vadim Pascua, Yueh-Ming Loo, Lucrezia Colonna, Sadie J. Van den Bogaerde, Jie An, Michael Gale, Karen Reue, Edward A. Fisher, Sina A. Gharib, Keith B. Elkon, Karin E. Bornfeldt

**Affiliations:** 1 Division of Metabolism, Endocrinology and Nutrition, Department of Medicine, University of Washington, Seattle, WA; 2 UW Medicine Diabetes Institute, University of Washington, Seattle, WA; 3 Division of Rheumatology, University of Washington, Seattle, WA; 4 Division of Pulmonary, Critical Care and Sleep Medicine, University of Washington, Seattle, WA; 5 Department of Anesthesiology and Pain Medicine, Northwest Metabolomics Research Center, University of Washington, Seattle, WA; 6 Department of Immunology, University of Washington, Seattle, WA; 7 Department of Human Genetics, David Geffen School of Medicine, University of California Los Angeles, Los Angeles, CA; 8 Division of Cardiology, Department of Medicine, New York University Grossman School of Medicine, New York, NY; 9 Department of Laboratory Medicine and Pathology, University of Washington, Seattle, WA

**Keywords:** Bis[monoacylglycerol]phosphates, enzymology/Enzyme mechanisms, glycerophospholipids, inflammation, lipotoxicity, macrophage, phospholipids/phosphatidic acid

## Abstract

Long-chain acyl-CoA synthetase 1 (ACSL1) catalyzes the conversion of long-chain fatty acids to acyl-CoAs. ACSL1 is required for β-oxidation in tissues that rely on fatty acids as fuel, but no consensus exists on why ACSL1 is induced by inflammatory mediators in immune cells. We used a comprehensive and unbiased approach to investigate the role of ACSL1 induction by interferon type I (IFN-I) in myeloid cells in vitro and in a mouse model of IFN-I overproduction. Our results show that IFN-I induces ACSL1 in macrophages via its interferon-α/β receptor, and consequently that expression of ACSL1 is increased in myeloid cells from individuals with systemic lupus erythematosus (SLE), an autoimmune condition characterized by increased IFN production. Taking advantage of a myeloid cell-targeted ACSL1-deficient mouse model and a series of lipidomics, proteomics, metabolomics and functional analyses, we show that IFN-I leverages induction of ACSL1 to increase accumulation of fully saturated phosphatidic acid species in macrophages. Conversely, ACSL1 induction is not needed for IFN-I’s ability to induce the prototypical IFN-stimulated protein signature or to suppress proliferation or macrophage metabolism. Loss of ACSL1 in IFN-I stimulated myeloid cells enhances apoptosis and secondary necrosis in vitro, especially in the presence of increased saturated fatty acid load, and in a mouse model of atherosclerosis associated with IFN overproduction, resulting in larger lesion necrotic cores. We propose that ACSL1 induction is a mechanism used by IFN-I to increase phosphatidic acid saturation while protecting the cells from saturated fatty acid-induced cell death.

Long-chain acyl-CoA synthetases (ACSLs) convert long-chain fatty acids to their fatty acyl-CoA derivatives (1), channeling fatty acids into various pathways, including glycerophospholipid and triglyceride synthesis and β-oxidation. The five mammalian ACSL isoforms have different fatty acid preferences and functions depending on in which cell type they are expressed and the fatty acid environment (2, 3). ACSL1 generates acyl-CoAs that are primarily used for β-oxidation in classical insulin target tissues, such as skeletal muscle, liver, and adipose tissue (3, 4, 5). However, in macrophages, ACSL1 is induced by a number of inflammatory stimuli, including toll-like receptor ligands, interferon-γ (IFNγ), type 1 interferons (IFN-I), and TNFα (6, 7, 8, 9, 10, 11). Small human cohorts also hint at myeloid cell ACSL1 expression being elevated in peripheral blood mononuclear cells (PBMCs) or whole blood in inflammatory states, such as type 1 diabetes or following a myocardial infarction or ischemic stroke (6, 12, 13, 14).

Why would inflammatory stimuli induce an enzyme that converts free fatty acids into acyl-CoAs in myeloid cells? Several explanations have been provided, including to increase production of inflammatory mediators (6, 10, 11, 15, 16), to boost β-oxidation (17), to allow phospholipid turnover (7), and to protect the lysosome from saturated fatty acid lipotoxicity (18)—but the exact reason is unknown. We used a comprehensive multi-omics approach combined with functional cell analyses and mouse studies to provide more definitive answers to the question above, focusing on IFN-I as an ACSL1 inducer. Our results suggest that IFN-I uses induction of ACSL1 as a mechanism to prevent lipotoxic effects of fatty acid overload in macrophages, in part, by making phosphatidic acid a reservoir for saturated fatty acids.

## Materials and Methods

### Mice

All animal studies were approved the Institutional Animal Care and Use Committee of the University of Washington. Wild-type C57BL/6J mice were from the Jackson Laboratory (Bar Harbor). Myeloid cell-targeted ACSL1-deficient (*Acsl1*^*fl/fl*^ *Lyz2-Cre*^*Tg/Tg*^) mice (ACSL1^M−/−^), backcrossed 10 generations onto the C57BL/6J background, have been described previously (6, 19). Controls were *Acsl1*^*wt/wt*^ *Lyz2-Cre*^*Tg/Tg*^ littermates (referred to as wild-type; WT). *Ifnar*^*−/−*^ mice on a pure C57BL/6J background were originally from J. Sprent (Scripps Institute, San Diego, CA), and *Irf3/7*^*−/−*^ mice (20) were originally provided by M.S. Diamond (Washington University School of Medicine, St. Louis, MO).

In some experiments, female ACSL1^M−/−^ mice and WT littermate controls received a single 0.5 ml intraperitoneal injection of pristane (tetramethylpentadecane; Sigma Aldrich) at 6–12 weeks of age. Female mice were used because SLE is more prevalent in women. Mice were euthanized at indicated times after pristane injection using Avertin (2,2,2,-tribromoethanol; Sigma Aldrich) or CO_2_. For isolation of blood leukocytes, whole blood was obtained by intracardiac puncture in EDTA-coated syringes. Cells were collected by centrifugation (400 *g* for 5 min at room temperature). Erythrocytes were lysed in sterile and endotoxin-free ACK buffer (0.15 M NH_4_Cl, 10 mmol/L KHCO_3_, 0.1 mmol/L Na_2_EDTA) for 5 min at room temperature, and the cells were centrifuged again as above to obtain leukocytes.

To study atherosclerosis, female *Ldlr*^*−/−*^ mice on the C57BL/6J background (The Jackson Laboratory, Strain #:002207) were irradiated and transplanted with bone marrow from ACSL1^M−/−^ mice (*Acsl1*^*fl/fl*^ *Lyz2-Cre*^*Tg/Tg*^) or littermate WT controls (*Acsl1*^*wt/wt*^ *Lyz2-Cre*^*Tg/Tg*^), as described (6). After 7 weeks of recovery, the mice were fed a low-fat semi-purified diet without added cholesterol, described previously (21), and injected i.p. with pristane or saline as described above. At the end of the study, aortas were dissected after in situ perfusion with PBS and then fixed in 10% phosphate-buffered formalin (MilliporeSigma). Aortas were opened longitudinally, from the heart to the iliac bifurcation, and an investigator blinded to the treatment groups quantified the extent of atherosclerosis *en face* after Sudan IV staining, as previously described (21). The aortic sinus was serial sectioned and stained with a Movat’s pentachrome stain. Lesion size was determined at three different distances from the appearance of all three aortic valves (0, 30 and 60 μm toward the aortic arch). The necrotic core area was defined as acellular areas with evidence of extracellular cholesterol accumulation without abundant matrix accumulation. All histological analyses were carried out by an investigator blinded to the study conditions.

### Isolation and stimulation of bone marrow-derived macrophages

Mouse femurs and tibias were cleaned and placed into a set of double Eppendorf tubes (a 0.5 ml tube with a hole in a 1.5-mL tube). Bone marrow was collected by centrifugation (8,000 *g* for 8 s) and then incubated in ACK buffer for 5 min to remove erythrocytes. After adding PBS to terminate lysis, leukocytes were collected by centrifugation at 300 *g* for 5 min. The cells were resuspended and plated in the presence of 30% L-conditioned medium (RPMI containing 30% L-conditioned medium [generated by culturing L cells, ATCC, CRL-2648, in high glucose DMEM containing 10% FBS, nonessential amino acids, and antibiotics for 3 days], 7% FBS and RPMI with 1% penicillin/streptomycin) and differentiated into bone marrow-derived macrophages (BMDMs), as described previously (6). At the seven-day point, media were changed and BMDMs were incubated for the indicated times in the presence of universal IFN-α (PBL; Piscataway, NJ), a hybrid interferon constructed from recombinant human IFN-α A/D [BglII], which activates mouse cells, or mouse recombinant IFN-β or vehicle (PBS/1% BSA). In some experiments, the cells were stimulated with resiquimod (R848, cat no 4536, Tocris Bioscience; 5 μg/ml) or vehicle (100% ethanol) or palmitate and oleate (Nu-Chek Prep, Inc.) at a 8:1 M ratio (palmitate:oleate) bound at a 1:3 M ratio to fatty acid-free BSA (BSA:fatty acid). Fatty acid-free BSA was used as control.

### Flow cytometry

Leukocyte populations were analyzed by specific antibodies purchased from BD Pharmingen or eBioscience: CD16/32 Fc Block (2.4G2); CD3-PE (17A2); B220-PerCPCy5.5 (RA3-6B2); Ly6C-PECy7 (AL-21); CD115-APC (AFS98); Ly6G-APCCy7 (1A8); CD11B-eFluor605NC (M1/70). Live (Fixable Viability Dye eFluor™ 450-negative), stained leukocytes were analyzed on a LSRII flow cytometer (BD Biosciences, Sa Jose, CA). A positive control for the viability dye was generated by heating cells to 60°C to induce cell death. Data collected (500,000 events per sample) were analyzed with FlowJo software (Tree Star, Inc). Negative controls incubated in the absence of antibody, negative control antibodies, and compensation controls were used to select positive cell populations by gating. In other experiments, BMDMs from ACSL1^M−/−^ mice and WT littermates were stimulated with 500 U/ml IFN-I for 24 h or PBS/1% BSA vehicle. Flow cytometry was used to assess levels of cell surface MHC II, CD80, and CD86 in live cells. See the Major Resources Supplement for further information on antibodies.

### Real-time PCR

Cells were lyzed in RLT buffer and RNA and cDNA were obtained (6). All samples were run in duplicates and all reactions were subsequently analyzed on agarose gels to exclude dimerization of primers and verify generation of a single reaction product per well. The primers used are listed in the Major Resources Supplement.

### Analysis of systemic factors

Plasma cholesterol, triglycerides, and IL-18 were analyzed as described previously (21, 22). Antibodies against single-stranded DNA (ssDNA) were measured using a previously published method (23). Plasma levels of mouse S100A8 and S100A9 were measured by ELISA (DuoSet ELISA, R&D Systems, cat no. DY8596).

### Analysis of glycerophospholipids by LC-MS/MS

WT and ACSL1-deficient BMDMs were stimulated with IFN-I (500 U/ml) or vehicle (PBS/1% BSA) for 24 h. The cells were then quickly harvested in PBS and frozen at −80°C. Glycerophospholipids were extracted from the cell pellets and analyzed, as described (24). In short, a commercial mixture of internal standards was used (SPLASH Lipidomix, from Avanti Polar Lipids, Inc.; 1 μl/sample) that contained 15:0/[^2^H_7_]18:1-PA (7 ng), 15:0/[^2^H_7_]18:1-PC (160 ng), 15:0/[^2^H_7_]18:1-PE (5 ng), 15:0/[^2^H_7_]18:1-PG (30 ng), 15:0/[^2^H_7_]18:1-PI (10 ng), and 15:0/[^2^H_7_]18:1-PS (5 ng). LC-MS/MS analysis was carried out in a system equipped with a HPLC system (Shimadzu) and a 4,000 QTRAP mass spectrometer (SCIEX), using a scheduled multiple reaction monitoring (MRM) method to detect molecular species containing combinations of common fatty-acyl chains. The precursor ions monitored were molecular ions [M-H]^−^ for all classes except PC, for which the acetate adducts [M + CH3COO]^−^ were monitored. The product ions analyzed after collision-induced decomposition were carboxylate anions corresponding to one of the acyl chains. A scheduled MRM window width of 450 s was used, centered on the following elution times: PA, 24.3 min; PC, 29 min; PE, 17 min; PG and BMP, 8 min; PI, 15 min; and PS, 23 min. Results were analyzed by using MultiQuant software (SCIEX) and are reported as the ratio between the integrated area of each analyte and the integrated area of the corresponding internal standard for each class.

### Global proteomics by LC-MS/MS

BMDMs from WT and ACSL1^M−/−^ mice (500,000 cells/well in 6-well tissue culture plates in RPMI containing 30% L-conditioned medium, 7% FBS and antibiotics) were stimulated with 500 U/ml IFN-I or vehicle (PBS/1% BSA) for 24 h. Macrophage peptide digests were prepared using the iST sample preparation kits according to the manufacturer’s instructions (PreOmics, Planegg/Martinsried). To measure the relative levels of the macrophage proteins, we used shotgun proteomics analysis as previously reported (25, 26, 27). Briefly, LC-ESI-MS/MS analyses were performed with an ultrahigh-resolution accurate mass Orbitrap Fusion Lumos Tribrid Mass Spectrometer (Thermo Fisher Scientific) coupled to a nanoACQUITY UPLC (Waters). Macrophage peptide digests (equivalent to 1 μg of protein) were desalted on a C-18 trap column (0.1 × 40 mm) at a flow rate of 3 μl/min for 7 min. They were then separated at a flow rate of 0.4 μl/min, using a C-18 analytical column (0.1 × 200 mm). Both the trap and analytical columns were packed in house with Magic C-18 reverse-phase resin (5 μm; 100 Å; Michrom Bioresources). The columns were kept at room temperature, and the peptides were separated using a multistep gradient as follows: 1–7% solvent B for 1 min; 7–25% solvent B for 105 min; 25–35% solvent B for 30 min; and 35–80% solvent B for 10 min. The column was subsequently washed for 5 min in 80% solvent B, 80–1% solvent B in 1 min, and then re-equilibrated in 1% solvent B for 19 min (solvent A, 0.1% formic acid in water; solvent B, 0.1% formic acid in acetonitrile).

The Orbitrap Fusion Lumos mass spectrometer was operated in positive ion mode and the peptide digests were analyzed in the data-dependent acquisition (DDA) mode, with 2,100 V of spray voltage and 300°C of ion transfer tube temperature for ionization. The acquisition method combined MS1 scans with DDA MS2 scans. For the MS1 scan, a scan range of m/z 350–1,800, an Orbitrap resolution of 120,000 (at m/z 200), a target automatic gain control (AGC) value of 400,000, and a maximum injection time of 50 ms were used. For the DDA MS2 scans, precursor ions with charge state of 2–5 were isolated using a 1.6 m/z window and an automatic scan range (starting from m/z 110), an Orbitrap resolution of 30,000 (at m/z 200), a target AGC value of 50,000, and a maximum injection time of 50 ms. Fragmentation was performed using higher energy collisional dissociation fragmentation with 30% collision energy with a stepped collision energy of 1.5%. Dynamic exclusion was set to detect a precursor ion once and the exclusion duration was set as 30 s. This approach facilitated extensive data-dependent MS/MS sampling and the generation of an adequate number of peptide-counts to reproducibly reflect relative peptide or protein abundance in macrophages.

MS/MS spectra were searched against the UniProtKB mouse database (20,190,114) using the Comet search engine (v2021.02.0) (28) with fixed Cys alkylation and variable Met oxidation modifications. One incomplete cleavage site was allowed in peptides for trypsin-restricted searches. The search results were further validated using PeptideProphet and ProteinProphet (29), using an adjusted probability of >0.90 for peptides and >0.95 for proteins. At least two peptides unique to the protein of interest had to be detected in at least 3 samples in any group. Requiring at least two unique peptides with a high confidence score markedly decreases the false-positive rate of protein identification (30). Each charge state of a peptide was considered a unique identification. Proteins identified by ≤3 unique peptides were inspected manually to verify the results. We used total spectral counting (31) to quantify the relative levels of the macrophage proteins.

### Metabolomics by LC-MS

BMDMs differentiated for 7 days were treated with IFN-I (500 U/ml) or vehicle (PBS/1% BSA) in fresh 30% L-conditioned medium for 24 h. The BMDMs were then washed 3 times with cold PBS, incubated in PBS on ice for 10 min, scraped and transfer into tubes. Cells (1.5 × 10^6^ cells) were pelleted and stored at −80°C until analyses.

For central carbon and glycolysis/TCA metabolomics, samples were maintained on wet ice throughout the processing steps. To each cell microtube, 500 μl extraction solvent (8:2 methanol:water containing 1 μM TCA internal standards [Cambridge Isotopes MSKTCA1]) was added. Samples were subjected to probe sonication set to level 4/40% power until completely homogenized. Samples were then incubated on ice for 10 min, vortexed, and cell debris was pelleted by centrifugation. 10 μl of the supernatant from each sample was removed to create a pooled sample for QC purposes. Next, 200 μl of the supernatant was transferred to an LC-MS autosampler insert and brought to dryness under a gentle stream of nitrogen at room temperature. Dried samples were reconstituted in 100 μl of 80/20 water/methanol for LC-MS analysis. A series of calibration standards (using Cambridge Isotopes MSKTCA1-US unlabeled TCA standards) were prepared along with samples to quantify metabolites. Calibration standards contained the same internal standard mixture and concentrations as the samples. Cell pellet protein quantitation was performed using the Pierce BCA Protein Assay kit (catalog no. 23227). Samples were analyzed on an Ion-LC-MS system consisting of an Agilent Infinity Lab II UPLC coupled with a 6545 QTOF mass spectrometer in negative ion mode, as previously described (32). For quantitative analysis, data were processed using MassHunter Quantitative analysis version B.07.00. Metabolites were normalized to the nearest isotope labeled internal standard and quantitated using 2 replicated injections of 5 standards to create a linear calibration curve with accuracy better than 80% for each standard. For semi-quantitative measurements, known compound peaks were manually integrated using Profinder v8.00 (Agilent Technologies), by matching the retention time (±0.1 min), mass (±10 ppm) and isotope profile (peak height and spacing) to authentic standards.

Acyl-carnitines were extracted from cell samples and derivatized using a protocol described by Giesbertz *et al.* (33). As above, 500 μl of 80%/20% methanol/water and 500 μl of mixture of stable isotope-labeled internal standards (SILISs) of known concentrations dissolved in 80%/20% methanol/water were added to the cell pellets. Samples were then vortexed for 10 s and sonicated for 30 min in an ice-bath. Samples were centrifuged at 10,000 *g* at 4°C for 10 min and 400 μl of the supernatant was collected and dried using a nitrogen drier. Samples were derivatized as follows: 500 μl of 95%/5% n-butanol/acetyl chloride was added, the samples were incubated at 60°C for 20 min, and then dried using nitrogen before reconstitution in 90%/10% methanol/water and vortexing (10 s). Finally, the samples were again centrifuged at 10,000 *g* and 4°C for 10 min and 300 μl of sample was transferred into LC-vials for LC-MS analysis. Acyl-carnitine species were quantified using an UPLC-MS/MS system composed of a Shimadzu Nexera X2 UPLC system connected to an AB Sciex 5500 QTrap MS equipped with an ESI source. The Shimadzu UPLC system was composed of a binary LC-30AD pump, DGU-20A 5R degasser, SIL-30AC autosampler set at 10°C (injection volume of 10 μl) and CTO-20AC temperature-controlled column compartment set at −40°C. The data were acquired using AB Sciex Analyst 1.6.3 software. All the molecules were ionized in positive ESI mode, and the mass analyzer was running in multiple-reaction monitoring (MRM) mode. Analytes were separated using reverse-phase (RP) chromatography on a Waters XSelect HSS T3 2.1 × 150 mm, 2.5 μm analytical column (Waters Part # 186006739). Mobile phases A and B were 10 mM ammonium acetate in 100% water plus 0.2% formic acid and 10 mM ammonium acetate in 5% water/5% methanol/90% acetonitrile plus 0.2% formic acid, respectively. The chromatography separation lasted 32 min total, from which the actual acyl-carnitines eluted from 6.8 min (free-carnitine) to 17.2 min (octadecanoyl-carnitine). ESI parameters were: Curtain Gas (CUR) = 30 psi, Collision Gas (CAD) = 9 L/min, Ion Spray Voltage (IS) = 4.5KV, Temperature (TEM) = 400°C, Ion Source Gas 1 (GS1) = 50 psi and Ion Source Gas 2 (GS2) = 40 psi. The assay targeted 67 MRMs, of which 54 were acyl-carnitines species and 13 were SILISs used to determine absolute concentrations of the measured analytes. Optimized MRM conditions for each acyl-carnitine species and spiked SILISs are listed in Metabolomics Data Supplement. For the species that had corresponding SILISs, the absolute concentration was determined directly by comparing MRM peak area of analyte to MRM peak area of corresponding SILIS of known concentration. For those acyl-carnitines without corresponding SILISs, their concentrations were determined indirectly by comparing peak areas of the analyte to that of the SILIS with the closest retention time to the analyte. MRM peak areas were measured using AB Sciex MultiQuant 3.03 software. From the cell samples, 22 acyl-carnitine species were measured. In order to monitor the system performance, an in-house QC sample (pooled human serum) was injected every 8 samples and used to monitor data reproducibility. In addition, a QC sample made from pooled study set was analyzed every 8 samples to determine the reproducibility of the run. For this study set of 24 cell extract samples, 4 QC runs were performed and the medium CV was 3.9%. From the 35 measured MRMs (22 analytes plus 13 SILISs) 23 had CV under 5%, 7 had CV 5–10% and 4 had CV 10–22%. The CVs were determined by measuring peak areas without any signal normalization. All the samples were acquired in one batch over a 20 h period. An example of total MRM ion chromatogram of the sample QC is shown in the Metabolomics Data Supplement.

### Measurements of OCR and ECAR

Oxygen consumption rate (OCR) and extracellular acidification rate (ECAR) were measured by using a Seahorse XF 96 instrument (Agilent). BMDMs were replated in 96-well Seahorse plates (6 × 10^4^ cells/well). After 2 days, the cells were treated with IFN-I (500 U/ml) or PBS/1% BSA vehicle for 24 h in 30% L-conditioned medium. A sensor cartridge was prepared and calibrated following the manufacturer’s protocol. The cells in Seahorse plates were washed once and equilibrated in XF base medium (pH 7.4) containing 2 mM glutamine (100 μl/well) for 30 min at 37°C. Monitoring OCR and ECAR was performed by consecutive injections of 10 mM glucose (A), 1.5 μM oligomycin (B), 1.5 μM FCCP plus 1 mM pyruvate (C) and 2.5 μM antimycin plus 1.25 μM rotenone (D) with 4 cycles of a mix-wait-measure between the injections. After the measurement was completed, the assay buffer was removed and the cell DNA content in each well was measured for normalization. First, 0.01% SDS in H_2_O (40 μl/well) was added and the plate was frozen at −80°C and thawed. Then, 40 μl of Hoechst staining solution was added per well (10 mM Tris, 1 mM EDTA and 4 μg/ml Hoechst in 1M NaCl), incubated for 1 h at 37°C and the fluorescence was measured.

### Membrane fluidity

We used a membrane fluidity kit from Abcam (ab189819) based on lipophilic pyrene probes that undergo excimer formation upon spatial interaction.

### Efferocytosis

Wildtype C57BL/6 thymocytes were labeled with CFSE (5-and 6-carboxyfluorescein diacetate succinimidyl ester; Invitrogen), UV irradiated (50 mJ/cm^2^) and incubated in DMEM + 1% BSA at 37°C for 4 h to undergo apoptosis (>65% cells were late apoptotic/secondary necrotic, as defined as Annexin V^+^ and PI^+^ cells). The apoptotic cells were added to BMDMs from WT and ACSL1^M−/−^ mice at different ratios (2:1; 4:1, and 8:1) for 20 and 40 min. The apoptotic cells were then washed off thoroughly with cold PBS. The BMDMs were subjected to trypsin-EDTA to harvest the BMDMs and to eliminate the residual surface-bound, non-internalized apoptotic cells. BMDMs were labelled with an anti-CD11b-APC antibody (clone M1/70; Biolegend) and analyzed by flow cytometry with the aid of a FACS Canto® (BD Biosciences).

In other experiments, ACSL1^M−/−^ and WT BMDMs were stimulated for 24 h with 500 U/ml IFN-I or vehicle (PBS/1% BSA), then washed once with PBS and co-incubated with dead Jurkat cells at 2:1 (Jurkat:BMDM) for 40 min. Cells were lifted with a cell scraper and stained for F4/80 and analyzed by flow cytometry.

### Proliferation

In vivo labeling of proliferating leukocytes and analysis by flow cytometry were performed using the Click-iT® Plus EdU Flow Cytometry Assay Kit (Life Technologies, cat# C10633). 5-ethynyl-2′-deoxyuridine (EdU) was dissolved in PBS to make a 8 mg/ml solution that was used to inject mice i.p. at 50 μg/g body weight, 15 h before harvest. Single cell suspension of the mouse bone marrow was obtained and stained for cell surface markers and the proliferating cells were stained following the kit’s protocol. The proliferating population of each leukocyte subpopulation was identified by flow cytometry on a 4-laser Canto RUO machine as EdU^+^ staining within specific cell populations. The flow cytometry data were analyzed by FlowJo software.

Cell cycle progression was measured in cultured BMDMs stimulated with 500 U/ml IFN-I or PBS/1% BSA vehicle. DNA content (S and G2/M phases) were measured by PI staining of fixed and permeabilized BMDMs and flow cytometry.

### Cell death assays

Apoptosis and secondary necrosis was measured in BMDMs stimulated in the presence 5% total FBS (including 15% L-conditioned medium) by using the RealTime-GLO™ Annexin V Apoptosis and Necrosis Assay (Promega) according to the manufacturer’s instructions. Cell death in real-time over longer periods of time was confirmed using an IncuCyte™ live cell system (Sartorius AG) and IncuCyte™ Cytotox Green Reagent (100 nM; cat no. 4633; Essen BioScience, Inc., Ann Arbor, MI). For these experiments, BMDMs (10 days after isolation) were incubated in RPMI, 2% FBS and 1% penicillin/streptomycin in the presence or absence of 150 μM fatty acid-free BSA complexed with 400 μM palmitate and 50 μM oleate, and/or 500 U/ml IFN-I or PBS/1% BSA vehicle.

Quantification of macrophages and TUNEL-positive macrophages in lesions of atherosclerosis was performed on sinus sections 40–50 μm from the appearance of all three aortic valves. The sinus paraffin sections were deparaffinized and then microwaved in 100 mM citrate buffer (pH 6) for 1 min for antigen retrieval. Sections were incubated with TUNEL solution (in situ cell death detection TMR red, Roche #12156792910) at 37°C for 1 h in a humidified container and washed. The sections were then incubated with a rat anti-Mac-2 antibody (0.5 μg/ml, Cedarlane CL8942AP) or rat IgG2a (0.5 μg/ml as negative control, Cedarlane CLCR2A00) in 3% goat serum, 1% BSA in PBS at 4°C overnight. After washing, the sections were incubated with a goat anti-rat Alexa488 conjugated secondary antibody in 1% BSA in PBS, washed, and mounted with Antifade mounting medium with DAPI (Vector Lab H-2000). Fluorescent images were captured by a fluorescent microscope (Keyence) and assessed by ImageJ.

### Immunoblot analysis

BMDMs were stimulated in the presence of 0–500 U/ml IFN-I for 24 h. Total cell lysates (10–50 μg) were loaded onto SDS/PAGE gels, separated, and transferred onto nitrocellulose membranes. Detection was accomplished by using the following primary antibodies: ACSL1 antibody (rabbit polyclonal #4047, 1:1,000 dilution; Cell Signaling Technology), β-actin antibody (mouse monoclonal, 1:10,000 dilution; Sigma-Aldrich). Specificity of the antibody was verified by lack of a band in ACSL1^M−/−^ BMDMs. Immunoblots for lipin 1 and lipin 2 were performed as previously described (34). HRP-conjugated secondary antibodies were used to detect the bands.

### Human studies

Characteristics of the human subjects are shown in Table 1. In the first SLE study, PBMCs were prepared from 20 healthy human donors and 46 SLE patients using Ficoll-Paque density gradient centrifugation, as previously described (35), and cDNA samples were generated. A replication study was performed on PBMCs isolated from 19 patients with SLE and 16 healthy control subjects. All SLE patients fulfilled the American College of Rheumatology 1982 revised criteria for the classification of SLE (36). All samples were collected with the University of Washington’s Institutional Review Board approval in compliance with the Helsinki Declaration.

**Table 1:** Clinical characteristics of the SLE discovery and replication cohorts

Demographic and clinical data	SLE	Controls
Discovery cohort		
No (% female)	46 (91.3)	20 (70)
Age (mean ± SD; years)	39.9 ± 13.3	33.7 ± 12.5
SLEDAI (mean ± SD)	3.4 ± 3.5	Not assessed
Replication cohort		
No (% female)	19 (94.7)	16 (68.8)
Age (mean ± SD; years)	37.2 ± 15.1	37.4 ± 14.7
SLEDAI (mean ± SD)	4.8 ± 3.9	Not assessed

A majority of participants with SLE were on hydroxychloroquine and prednisone (2–60 mg/day) therapy.

SLEDAI, Systemic Lupus Erythematosus Disease Activity Index.

### Statistical analyses and bioinformatics

GraphPad Prism 10.3.0 (GraphPad Software, Inc) was used for statistical analyses. The D'Agostino & Pearson test was used to determine if data were normally distributed. Statistical outliers were identified by ROUT Q = 0.1%. Unpaired *t* test was used when comparing two groups of normally distributed data. Mann-Whitney *U* test was used for non-parametric data comparison between two groups. One-way ANOVA followed by Tukey’s multiple comparison tests was used to analyze normally distributed data from more than 2 groups, and Kruskal-Wallis followed by Dunn’s multiple comparison tests were used for non-normally distributed data. Two-way ANOVA followed by Tukey’s multiple comparison tests or Šídák's multiple comparisons test was used as indicated in the Original Data Supplement. Probabilities of less than 0.05 were considered statistically significant. All *P* values are based on two-tailed analyses. See the Original Data Supplement for further details on statistical analyses for all figures.

Proteomics data were analyzed using DESeq2 software (R environment) (37), and unpaired two-tailed *t* test was applied to lipidomics and metabolomics data to identify differentially expressed features. Lipidomics, proteomics and metabolomics results were corrected for multiple comparisons using Benjamini-Hochberg adjusted *P* values. Proteomics pathway enrichment analysis was performed using Ingenuity Pathway Analysis (Qiagen).

## Results

### Myeloid cell ACSL1 is induced by IFN-I and correlates with IFN-I scores in individuals with SLE

**Fig. 1:**
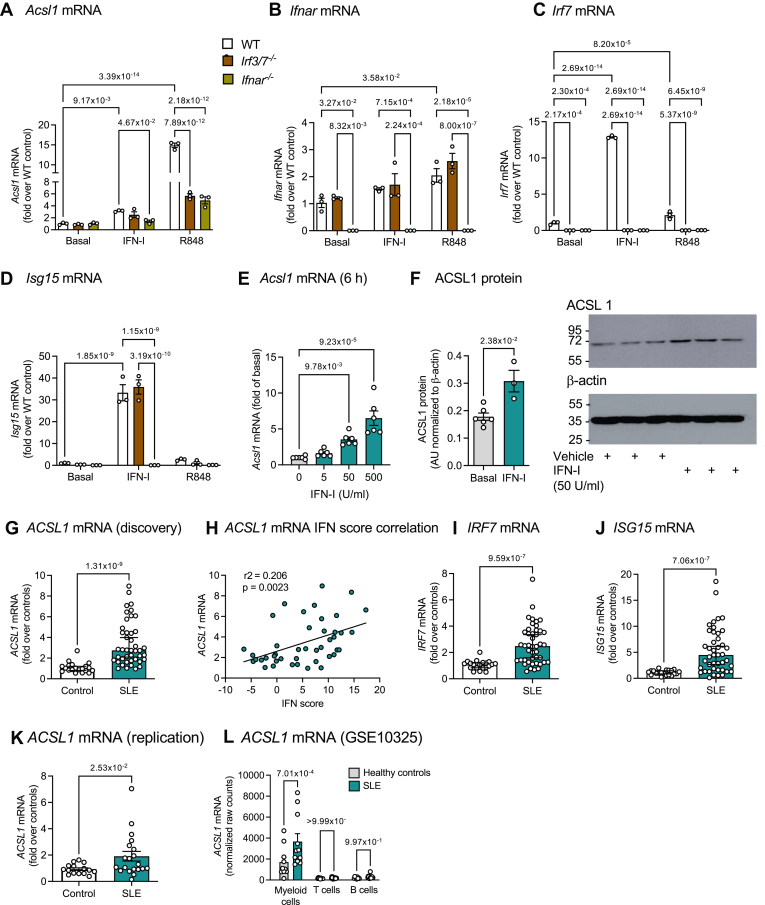
ACSL1 is induced by IFN-I via IFNAR and by SLE in myeloid cells. A–D: Mouse BMDMs prepared from *Irf3/7*^*−/−*^ mice, *Ifnar*^*−/−*^ mice and wildtype C57BL/6J (WT) controls and were stimulated with 50 U/ml IFN-I or the TLR7/8 ligand R848 (5 μg/ml) or vehicle for 6 h. *Acsl1, Ifnar, Irf7 and Isg15* mRNA levels were measured by real-time PCR. E, F: Mouse BMDMs from C57BL/6J mice were stimulated with 0, 5, 50 or 500 U/ml IFN-I for 6 h (E) or with 50 U/ml IFN-I or vehicle (PBS/1% BSA) for 24 h (F). *Acsl1* mRNA was measured by real-time PCR and ACSL1 protein levels were evaluated by immunoblot. The intensity of the ACSL1 band was normalized to that of β-actin and was expressed as fold over basal. G: PBMCs from subjects with SLE (46 subjects) and healthy controls (20 subjects) were analyzed for mRNA levels of *ACSL1* by real-time PCR. H: Correlation between *ACSL1* and IFN score in the same SLE samples by Pearson correlation. (I, J) PBMCs from subjects with SLE and controls in panel (G) were analyzed for *IRF7* (I) and *ISG15* (J) mRNA by real-time PCR. K: *ACSL1* mRNA was measured in PBMCs from a replication study on 16 control subjects and 19 subjects with SLE. L: An existing array data set (GSE10325) was probed for *ACSL1* mRNA levels in myeloid cells, CD4^+^ T cells, and CD19^+^ B cells from subjects with SLE and controls. Results are presented as scatter plots and mean ± SEM. Original data, statistical analyses, and numbers/group are shown in the Original Data Supplement. AU, arbitrary unit.

We first investigated whether IFN-I induces ACSL1 via its interferon-α/β receptor (IFNAR) in mouse BMDMs. Consistent with a previous study (10), IFN-I induced increased *Acsl1* mRNA levels (Fig. 1A). This effect was dependent on IFNAR (Fig. 1A, B). Moreover, the effect of IFN-I was direct and not mediated by interferon regulatory factor 3 or 7 (IRF3 and IRF7) (Fig. 1A–C). The TLR7/8 ligand R848 induced a larger increase in *Acsl1* mRNA levels, which was dampened by two thirds in BMDMs lacking IFNAR or IRF3/7, consistent with the known role of IRF7 activation and subsequent IFN-I production following TLR7/8 activation (38). Approximately one third of the *Acsl1* induction by R848 was mediated by a pathway independent of IFN-I (Fig. 1A–C). One possible transcription factor involved in this pathway could be activator protein 1 (38), as we have previously shown recruitment of the activator protein 1 constituent c-Jun to the *Acsl1* promoter in mouse macrophages stimulated with lipopolysaccharide (7). As expected, IFN-I-induced expression of a typical IFN-stimulated gene (interferon-stimulated gene 15; *Isg15*) was also dependent on IFNAR but not IRF3/7 (Fig. 1D). IFN-I induced a dose-dependent increase in *Acsl1* mRNA levels with a statistically significant effect observed at 50 U/ml (corresponding to 0.5 ng/ml) 6 h after stimulation (Fig. 1E). Consequently, 50 U/ml IFN-I increased ACSL1 protein levels at 24 h (Fig. 1F).

The autoimmune disease SLE is associated with elevated circulating IFN-I levels and IFN gene expression signatures, which correlate with disease severity (39, 40, 41, 42, 43, 44). We reasoned that if IFN-I induces ACSL1, individuals with SLE should exhibit increased expression levels of ACSL1 in myeloid cells. To address this question, we isolated PBMCs from a discovery cohort consisting of 46 individuals with SLE and 20 healthy controls (Table 1), and measured *ACSL1* mRNA as well as mRNA levels of known IFN-I-stimulated genes. *ACSL1* mRNA levels were significantly elevated in PBMCs from individuals with SLE, as compared with healthy controls (Fig. 1G). Moreover, *ACSL1* mRNA levels correlated positively with the patients’ IFN score (Fig. 1H), calculated from the combined expression of three prototypical IFN-responsive genes (*MX1*, *CXCL10* and *EIF2AK2*), as described previously (35). The interferon signature genes *IRF7* and *ISG15* were also elevated in individuals with SLE, as expected (Fig. 1I, J). To strengthen these findings, we collected PBMCs from an independent replication cohort consisting of 19 subjects with SLE and 16 controls (Table 1). Consistent with the discovery cohort, individuals with SLE exhibited elevated levels of *ACSL1* mRNA in their PBMCs (Fig. 1K). In addition, we mined expression profiling arrays from a previous study in which PBMCs from 14 subjects with SLE and matched controls were sorted into myeloid cells, CD4^+^ T cells and CD19^+^ B cells (45). The results show that myeloid cells is the primary PBMC cell population in which ACSL1 is upregulated in response to SLE (Fig. 1L).

Together, our findings provide evidence that IFN-I induces ACSL1 expression in murine myeloid cells via IFNAR and that SLE, an autoimmune condition characterized by IFN-I overproduction, shows increased expression of *ACSL1* in myeloid cells, showing human relevance.

### IFN-I induces an increase in fully saturated phosphatidic acid species through ACSL1

**Fig. 2:**
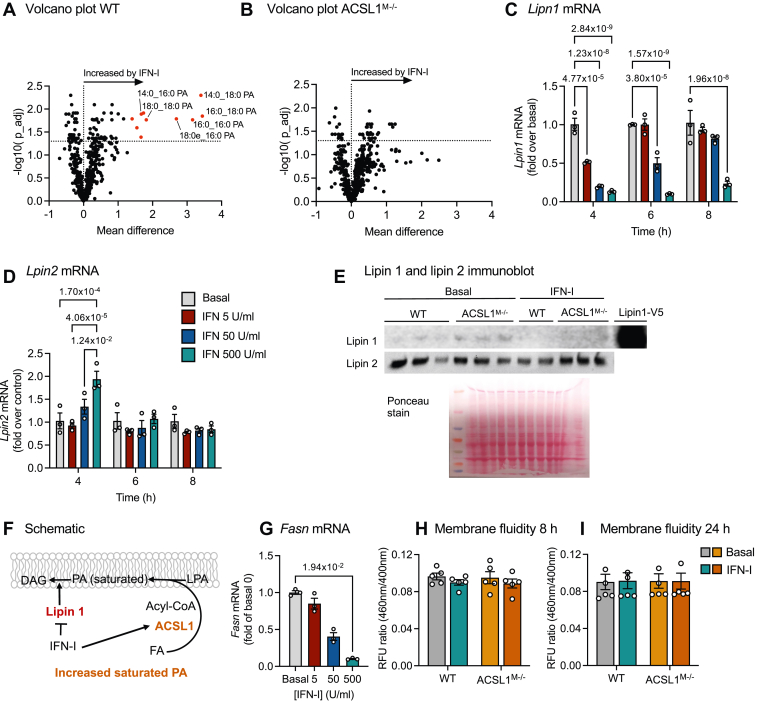
IFN-I increases saturated phosphatidic acid species via ACSL1 in macrophages. A, B: BMDMs from ACSL1^M−/−^ mice and WT littermate controls were stimulated for 24 h with 500 U/ml IFN-I or vehicle (PBS/1% BSA). A phospholipidomic screen was used to assess alterations in 500 identified glycerophospholipids (see Phospholipidomics Data Supplement). A: Differences in relative abundance in phospholipid species in WT BMDMs stimulated with IFN-I shown in a volcano plot. Horizontal dotted line indicates an adjusted *P* value cut-off of < 0.05. *Red* symbols indicate fully saturated glycerophospholipid species with a difference >1. B: Differences in relative abundance in phospholipid species in ACSL1^M−/−^ BMDMs shown in a volcano plot. No differences in saturated phosphatidic acid (PA) species reached statistical significance. C: *Lipn1* mRNA levels were measured in WT BMDMs stimulated with indicated concentrations of IFN-I for 4, 6 or 8 h. D: *Lpin2* mRNA levels in the same samples. E: Immunoblot of lipin 1 and lipin 2 after 24 h stimulation with 500 U/ml IFN-I or vehicle. The Ponceau-stained membrane is shown as a protein loading control (n = 2–3). V5 epitope-tagged lipin 1 expressed in HEK 293 cells served as a positive control. The experiment was repeated 2 times at different time points with similar results. (F) Schematic representation of the effect of IFN-I. G: *Fasn* mRNA levels were measured in the cells in (C, D) 6 h after IFN-I stimulation. H, I: Membrane fluidity was measured at the indicated times after IFN-I stimulation (500 U/ml) or vehicle (PBS/1% BSA vehicle). Results are presented as volcano plots (A, B) or scatter plots and mean ± SEM. Original data, statistical analyses, and numbers/groups are shown in the Original Data Supplement and in the Phospholipidomics Data Supplement.

In order to gain a better understanding of the role of ACSL1 induction by IFN-I, we undertook an unbiased lipidomics screen of 500 glycerophospholipids in BMDMs from mice lacking ACSL1 in myeloid cells (ACSL1^M−/−^ mice) and WT littermate controls. IFN-I had marked effects on the global glycerophospholipidome both in WT and ACSL1-deficient BMDMs (see the Phospholipidomics Data Supplement). The most marked difference between IFN-I stimulation in the two groups of BMDMs was observed in phosphatidic acid (PA) species. IFN-I induced a significant increase in PA species containing fully saturated acyl-chains (14:0, 16:0 and 18:0) (Fig. 2A). IFN-I failed to induce a significant increase in saturated PA species in ACSL1-deficient BMDMs (Fig. 2B), suggesting that induction of ACSL1 by IFN-I serves to increase saturated PA species in macrophages. Once formed, PA can be converted to diacylglycerol (46) through the action of PA phosphatases (lipins) (47, 48, 49). Since IFN-I caused an increase in saturated PA species, we reasoned that in addition to inducing ACSL1, lipins might be inhibited by IFN-I, thus further enhancing saturated PA accumulation. Indeed, *Lipn1* mRNA levels were markedly suppressed by IFN-I in a dose-dependent manner at 4–8 h after IFN-I stimulation (Fig. 2C), while *Lipn2* mRNA levels were not suppressed and even increased (Fig. 2D). We confirmed that lipin 1, but not lipin 2, protein levels were reduced by IFN-I at 24 h (Fig. 2E). Thus, IFN-I relies on two strategies to increase saturated PA species—induction of ACSL1 and suppression of lipin 1 (Fig. 2F). The increased levels of fully saturated PA species is unlikely to be due to increased de novo fatty acid synthesis because fatty acid synthase (*Fasn*) gene expression was markedly suppressed by IFN-I in macrophages (Fig. 2G), consistent with previous studies (68).

Because Ruiz *et al.* (50) showed that silencing ACSL1 in HEK293 cells protects against the membrane rigidifying effects of palmitate, we measured membrane fluidity in BMDMs. However, we found no effect of IFN-I or ACSL1-deficiency at two different time-points (Fig. 2H, I). Together, these results suggest that IFN-I-induced ACSL1 expression does not lead to an overall rigidifying effects on macrophage membranes, despite the increase in fully saturated PA species.

### IFN-I does not need ACSL1 for induction of prototypical IFN-signatures or effects on efferocytosis, proliferation, or macrophage metabolism

**Fig. 3:**
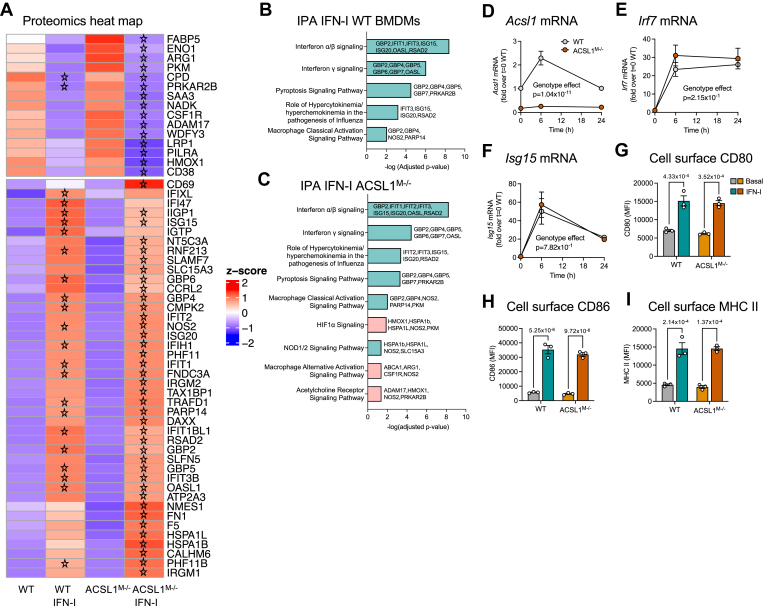
ACSL1-deficiency does not alter prototypical IFN-I-induced signatures in macrophages. A: Heat map showing significant changes by 500 U/ml IFN-I (24 h stimulation) in WT and ACSL1^M−/−^ BMDMs (adjusted *P* value < 0.1). Stars indicate the most statistically significant changes by IFN-I (adjusted *P* value < 0.05), as compared with vehicle-treated WT or ACSL1^M−/−^ cells, respectively. B: IPA of significantly altered pathways by IFN-I in WT BMDMs. C: IPA of significantly altered pathways by IFN-I in ACSL1^M−/−^ BMDMs (adjusted *P* value < 0.05 in B and C). Light teal bars indicate positive z scores (pathways activated by IFN-I stimulation) while light pink bars indicate negative z-scores (pathways deactivated by IFN-I). D–F: *Acsl1*, *Irf7* and *Isg15* mRNA levels in BMDMs stimulated for indicated times with 500 U/ml IFN-I. G–I: Cell surface CD80, CD86, and MHC II were analyzed by flow cytometry after 24 h stimulation with 500 U/ml IFN-I or vehicle (PBS/1% BSA). Results are presented as mean ± SEM. Original data, statistical analyses, and numbers/groups are shown in the Original Data Supplement and the Proteomics Data Supplement.

To investigate whether the ACSL1-dependent glycerophospholipid alterations contribute to prototypical IFN-I functions, we took a comprehensive approach. We first analyzed the effects of IFN-I on global changes in the macrophage proteome by unbiased shot-gun mass spectrometry. IFN-I markedly induced prototypical IFN-stimulated proteins in both ACSL1^M−/−^ BMDMs and BMDMs from WT littermate controls, including ISG15, IFIH1, IFIT3B and IFIT1BL1 (Fig. 3A). Consistently, Ingenuity Pathway Analysis (IPA) revealed that five pathways were significantly enriched by IFN-I stimulation both in WT and ACSL1^M−/−^ BMDMs; IFNα/β signaling, IFNγ signaling, pyroptosis signaling pathway, role of hypercytokinemia/hyperchemokinemia in the pathogenesis of influenza, and macrophage classical activation signaling pathway (Fig. 3B, C). Four additional pathways were significantly modified by IFN-I in ACSL1^M−/−^ BMDMs; a NOD1/2 signaling pathway was enriched, while HIF1α signaling, macrophage alternative activation signaling pathway, and acetylcholine receptor signaling pathway were repressed. Several of those pathways shared the proteins NOS2, HMOX1 and the heat shock proteins HSPA1B and HSPA1L. Thus, ACSL1-deficiency has modest effects on the macrophage proteome. The main protein reduced in BMDMs from ACSL1^M−/−^ mice was ACSL1 (adjusted *P* values 3.62 × 10^−13^ in IFN-I-stimulated cells and 5.19 × 10^−15^ in vehicle-treated cells).

Consistent with the lack of differences in prototypical IFN-induced proteins between ACSL1^M−/−^ and WT BMDMs, ACSL1-deficiency also did not alter mRNA levels of the IFN-I-induced genes *Irf7* and *Isg15* after IFN-I stimulation (Fig. 3D, F). To further confirm that typical IFN-stimulated proteins were not altered by ACSL1-deficiency, we analyzed cell surface CD80 and CD86 (co-stimulatory molecules involved in T cell activation) and MHC II (major histocompatibility complex involved in antigen presentation) in BMDMs. As expected, IFN-I induced markedly increased cell surface levels of these molecules (Fig. 3G–I). There were no differences between WT and ACSL1^M−/−^ BMDMs.

**Fig. 4:**
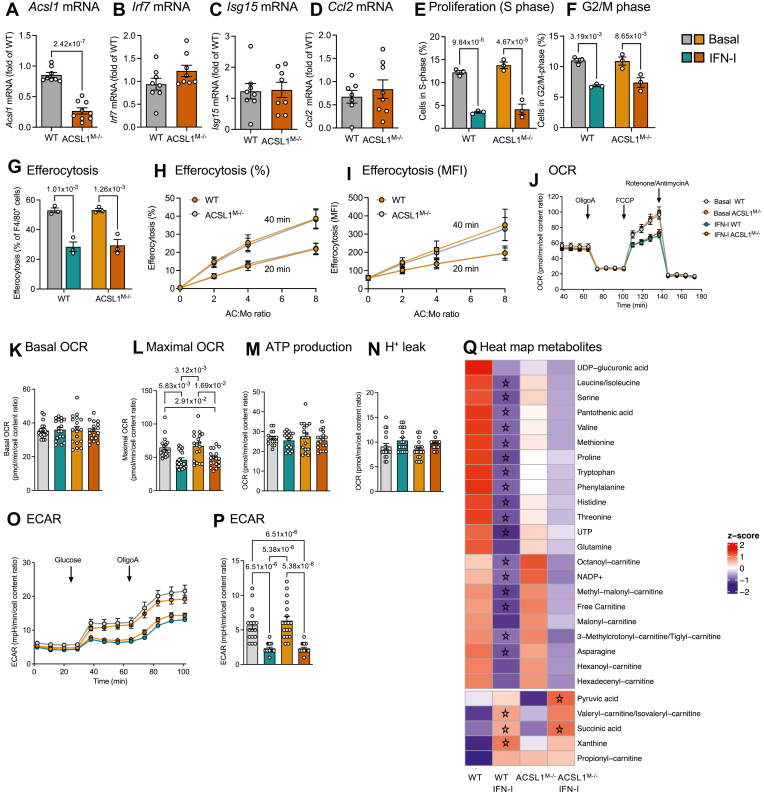
Myeloid cell ACSL1-deficiency does not alter IFN-I-induced genes, suppression of proliferation, or efferocytosis, and has subtle effects on metabolism. A–D: ACSL1^M−/−^ mice and WT littermate controls were injected i.p. with pristane. Peritoneal exudate cells were collected 2 weeks later and used for analysis by real-time PCR of *Acsl1* mRNA (A), *Irf7* mRNA (B), *Isg15* mRNA (C), and *Ccl2* mRNA (D). E, F: Cell cycle phases were analyzed by PI staining and flow cytometry in BMDMs stimulated with 500 U/ml IFN-I or vehicle (PBS/1% BSA) for 24 h. G: Efferocytosis was analyzed in BMDMs incubated with 500 U/ml IFN-I or vehicle for 24 h, then co-incubated with dead Jurkat cells at a 2:1 (Jurkat:BMDM) ratio for 40 min. Cells were lifted with a cell scraper and stained for F4/80 and analyzed by flow cytometry. H, I: Apoptotic mouse thymocytes were added to BMDMs from wildtype and ACSL1^M−/−^ mice at indicated ratios and times. BMDMs were labelled with an anti-CD11b-APC antibody and analyzed by flow cytometry. H: Efferocytosis expressed as % of CD11b + cells that had efferocytosed thymocytes. I: Efferocytosis expressed as CFSE mean fluorescence intensity. J: Oxygen consumption rate (OCR) measured in real time by an Agilent Seahorse XF Analyzer in BMDMs preincubated in the presence of 500 U/ml IFN-I or vehicle for 24 h. K: Basal OCR. L: Maximal OCR. M: Calculated ATP production. N: Calculated H^+^ leak. O: Extracellular acidification rate (ECAR) in the same cells used for OCR analysis. P: Calculated ECAR. Q: Heat map showing nominally significant metabolites analyzed by targeted mass spectrometry (adjusted *P* value < 0.1). Stars indicate the most statistically significant changes by IFN-I (adjusted *P* value < 0.05), as compared with basal WT or ACSL1^M−/−^, respectively. Original data, statistical analyses, and numbers/group are shown in the Original Data Supplement and in the Metabolomics Data Supplement.

The above experiments ruled out marked effects of ACSL1-deficiency on canonical IFN-stimulated responses in cultured BMDMs. To investigate the effects of myeloid cell-targeted ACSL1-deficiency in vivo, we injected ACSL1^M−/−^ mice and WT littermate controls i.p. with pristane, used to model the IFN-I signature in SLE (51). The IFN signature in pristane-injected mice is mediated by IFNAR (52). Myeloid cell-targeted ACSL1-deficiency resulted in an almost 70% reduction of *Acsl1* mRNA in the peritoneal exudate cells (a population also containing some non-myeloid cells), but consistent with the in vitro studies, did not affect *Irf7* and *Isg15* mRNA levels or *Ccl2* mRNA levels in those cells (Fig. 4A–D).

We next investigated several other functional effects of IFN-I in cultured BMDMs. IFN-I is known to suppress proliferation of macrophages (53), which was observed also in our studies (Fig. 4E, F). However, ACSL1-deficiency did not alter the suppressive effect of IFN-I on cell cycle progression. The ability of myeloid cells to clear apoptotic cells is impaired in SLE (54), but ACSL1-deficiency did not impair efferocytosis of apoptotic cells (Fig. 4G–I), consistent with our previous studies (55).

Since IFN-I has been shown to suppress oxygen consumption rate (OCR) and glycolysis in macrophages (56), we next investigated the effects of IFN-I on OCR and glycolysis in BMDMs. IFN-I suppressed both maximal OCR and extracellular acidification rate (ECAR, a marker of glycolysis), as in previous studies (56), but ACSL1-deficiency had no marked effect on those parameters (Fig. 4J–P). To further explore cellular metabolism, we performed several metabolomics experiments (Fig. 4Q). Consistent with the reduced OCR and glycolysis in BMDMs stimulated with IFN-I, IFN-I caused reductions in free carnitine and some acyl-carnitines but also in other metabolites and amino acids. One notable exception was an increase in succinate in IFN-I-stimulated WT and ACSL1^M−/−^ BMDMs, consistent with the known block in the TCA cycle in macrophages stimulated with LPS (57). The effects of IFN-I on macrophage metabolites were dampened by ACSL1-deficiency, suggesting subtle effects of ACSL1-deficiency on macrophage metabolism. One exception was the significant increase in pyruvate in IFN-I-stimulated ACSL1^M−/−^ BMDMs (Fig. 4Q). These changes did not, however, translate to changes in OCR or ECAR.

### ACSL1 serves to protect myeloid cells from apoptosis and secondary necrosis in vitro and in vivo

**Fig. 5:**
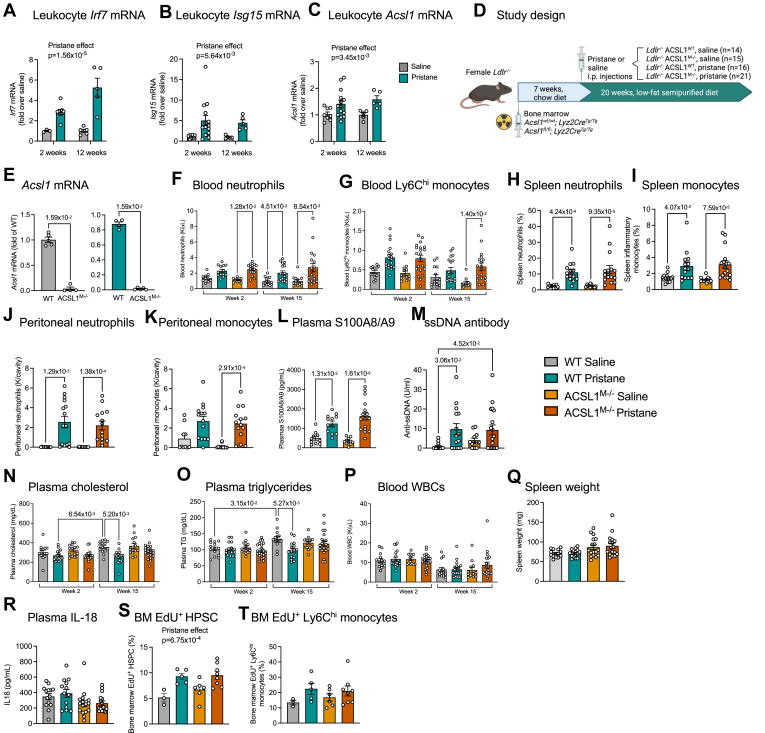
Myeloid cell ACSL1-deficiency does not alter myeloid cell numbers or markers of myeloid cell activation in a pristane mouse model of IFN-I overproduction. A–C: Blood leukocytes were collected from C57BL/6J mice 2 and 12 weeks after pristane injection. Levels of mRNA were measured by real-time PCR. A: Leukocyte *Irf7* mRNA. B: Leukocyte *Isg15* mRNA. C: Leukocyte *Acsl1* mRNA. D: Study design. E: *Acsl1* mRNA levels in BMDMs from transplanted mice at the end of the study. F: Blood neutrophil numbers at 2 and 15 weeks after pristane injection. G: Blood Ly6C^hi^ monocyte numbers at 2 and 15 weeks. (H) Spleen neutrophils at the end of the study. I: Spleen inflammatory monocytes gated on single cells/live cells/CD3-CD19^-^/Siglec F^-^/CD11b^+^/CD11c^-^/Ly-6G-Ly6C^++^. J: Peritoneal neutrophil number. K: Peritoneal monocyte number. L: Plasma S100A8/A9 levels measured by ELISA at the end of the study. M: Plasma ssDNA antibodies at the end of the study. N: Plasma cholesterol at 2 and 15 weeks after pristane injection. O: Plasma triglycerides at 2 and 15 weeks after pristane injection. P: Blood WBC numbers at 2 and 15 weeks. (Q) Spleen weight. R: Plasma IL-18 levels measured by ELISA at the end of the study. S: Bone marrow proliferating (EdU^+^) HSPCs. T: Bone marrow proliferating (EdU^+^) Ly6C^hi^ monocytes. Results are presented as scatter plots with mean ± SEM. Original data, statistical analyses and numbers/group are shown in the Original Data Supplement.

To further investigate the function of myeloid cell ACSL1 in vivo, we again turned to the pristane mouse model. As with human SLE, mice injected with pristane develop an IFN signature (51, 58), which we show includes increased leukocyte *Acsl1* mRNA levels (Fig. 5A–C). Because SLE is associated with an increased risk of cardiovascular disease (59) and because we had previously shown that myeloid-targeted ACSL1-deficiency results in smaller early lesions of atherosclerosis due to reduced macrophage accumulation in a mouse model of type 1 diabetes (another autoimmune disease associated with increased IFN signaling) (6, 25), we used LDL receptor-deficient (*Ldlr*^*−/−*^) mice to allow analysis of atherosclerosis. In short, female *Ldlr*^*−/−*^ mice were irradiated and then transplanted with bone marrow from ACSL1^M−/−^ mice and WT littermate controls. After 7 weeks of recovery, the mice were injected with pristane or saline, as shown in Fig. 5D. BMDMs isolated from mice transplanted with ACSL1^M−/−^ bone marrow at the end of the 20 weeks study showed a near complete loss of *Acsl1* mRNA expression, as expected (Fig. 5E). Pristane injected mice exhibited the known neutrophilia and monocytosis (51), which was observed not only in circulation, but also in the spleen and peritoneal cavity (Fig. 5F–K). Consistent with neutrophil activation, plasma levels of S100A8/A9 were elevated in pristane-injected mice (Fig. 5L). At the end of the study, plasma levels of single-standed DNA antibodies were elevated (Fig. 5M), consistent with autoimmunity in this model (51). Myeloid-targeted ACSL1-deficiency had no marked effects on any of these parameters.

Pristane had only modest or no effects on plasma lipids, total blood leukocyte numbers, spleen weight or plasma IL-18 levels (Fig. 5N–R). However, mice injected with WT bone marrow exhibited a modest reduction in plasma lipids at the 15 weeks time-point. Pristane increased proliferation of bone marrow hematopoietic stem and progenitor cells (HSPCs), but myeloid ACSL1-deficiency did not have an effect in those cells or downstream bone marrow myeloid cells (Fig. 5S–T) consistent with the lack of in vitro effects on proliferation. These findings further strengthen the conclusion that myeloid ACSL1 is not required for the effects of IFN-I on well-established IFN-stimulated responses.

**Fig. 6:**
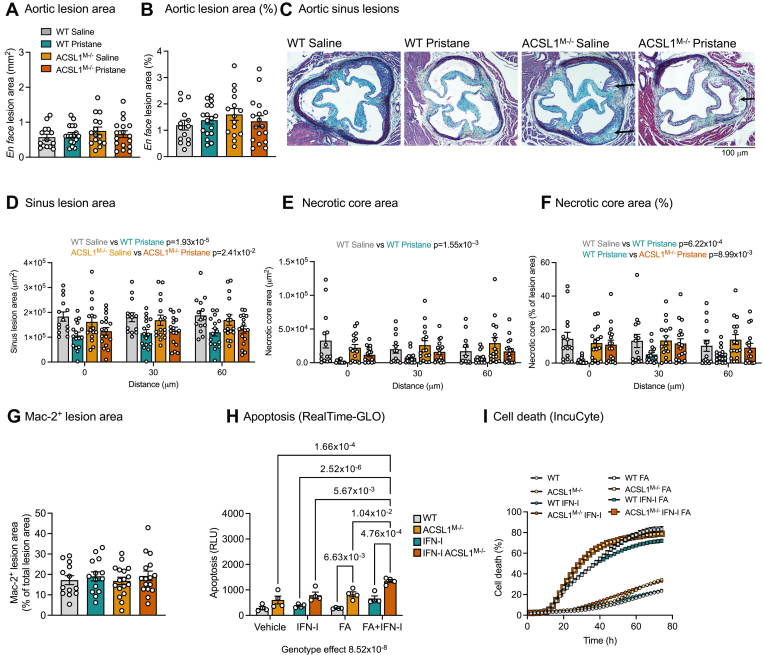
Myeloid cell ACSL1-deficiency results in increased cell death in vitro and in lesions of atherosclerosis. The study design for panel (A–G) is shown in Figure 5D. A: Total aortic lesion area measured *en face* as Sudan IV-positive area at the end of the study. B: Aortic lesion area measured *en face* as Sudan IV-positive area % of total aortic area. C: Representative cross sections of aortic sinus lesions stained by Movat’s pentachrome. Examples of necrotic cores are indicated by arrows. Scale bar = 100 μm. D: Aortic sinus lesion area quantified at 0, 30 and 60 μm from the appearance of all three aortic valves towards the aortic arch. E: Total sinus lesion necrotic core area quantified at 0, 30 and 60 μm. F: Sinus necrotic core area as % of lesion area quantified at 0, 30 and 60 μm. G: Mac-2-positive sinus lesion area at 40–50 μm. H: Apoptosis was measured by the RealTime-GLO™ Annexin V Apoptosis and Necrosis Assay (Promega) in WT and ACSL1^M−/−^ BMDMs stimulated with and without fatty acids (FA) and IFN-I (500 U/ml; n = 4) for 3 h. I: BMDM cell death was analyzed in real-time over 74 h by an IncuCyte™ instrument in WT or ACSL1^M−/−^ cells stimulated with IFN-I (500 U/ml) or vehicle (PBS/1% BSA) and with or without fatty acids (FA). Results are presented as scatter plots and/or mean ± SEM. Original data, statistical analyses and numbers/group are shown in the Original Data Supplement.

We next analyzed atherosclerosis in the four groups of mice. Despite the marked neutrophilia and monocytosis, pristane did not increase atherosclerosis measured as *en face* Sudan IV staining of the aorta and significantly reduced the cross-sectional lesion size in the aortic sinus in both WT and ACSL1^M−/−^ mice (Fig. 6A–D), perhaps because of the modest reduction in plasma cholesterol. Pristane also caused reduced necrotic cores in WT mice. Strikingly, myeloid-targeted ACSL1-deficiency resulted in a marked increase in lesion necrotic core area only in pristane-treated mice, i.e., under increased IFN-I load (Fig. 6E, F). However, myeloid ACSL1-deficiency had no significant effect on lesion macrophage content (Fig. 6G), and there were no detectable increases in TUNEL-positive macrophages in the ACSL1^M−/−^ mice (WT saline 4.0 ± 1.0 TUNEL-positive Mac2-positive cells/sinus lesion section; WT pristane 4.1 ± 1.2; ACSL1^M−/−^ saline 5.2 ± 1.2 and ACSL1^M−/−^ pristane 3.1 ± 0.8 cells), perhaps due to the late stage of the lesions.

To investigate if ACSL1-deficiency makes macrophages more susceptible to apoptosis and secondary necrosis, we set up several complementary in vitro experiments. These experiments revealed that ACSL1-deficient macrophages exposed to fatty acids exhibited a higher level of apoptosis than WT macrophages after 3 h of exposure, an effect exacerbated in the presence of IFN-I (Fig. 6H). Moreover, fatty acid exposure markedly increased secondary necrosis measured in real-time beyond 24 h, and ACSL1-deficient macrophages underwent secondary necrosis earlier than WT macrophages (Fig. 6I). Induction of ACSL1 by IFN-I in WT cells protected from fatty acid-mediated secondary necrosis, while this effect was lost in ACSL1-deficient cells (Fig. 6I).

## Discussion

Our study is consistent with the proposal that ACSL1 induction is a mechanism leveraged by IFN-I to increase PA saturation while protecting the cells from saturated fatty acid-induced cell death. However, the increased PA saturation is unlikely to affect IFN-I-induced signaling or induction of the prototypical interferon signature response, because no obvious impairment of IFN-I’s effects on interferon-induced pathways were observed in ACSL1-deficient myeloid cells. Likewise, the effects of IFN-I on cell metabolism (oxygen consumption and glycolysis), proliferation, and efferocytosis were unaffected by the loss of ACSL1. Moreover, no impairment in the effects of pristane, a hydrocarbon oil that causes overproduction of type 1 IFN α and β (51), on induction of IFN-induced genes, myelopoiesis, plasma S100A8/A9 and IL-18 levels, or ssDNA antibody production were observed in mice lacking ACSL1 in myeloid cells.

PA, an anionic lipid, is mostly present on the inner leaflet of the plasma membrane and on the ER membrane. Cholesterol strongly prefers to associate in membranes with anionic lipids over neutral lipids and saturated acyl chains over unsaturated (60). It is therefore possible that the increase in fully saturated PA species by IFN-I serves to localize free cholesterol to particular membrane domains. In this context, it is interesting that IFN-I protects macrophages from the pore forming cholesterol-dependent cytolysins secreted by bacteria by reducing a small “accessible” membrane cholesterol pool (61). Restricting cholesterol accessibility by increasing its binding to saturated PA could serve as a strategy to protect the cell from succumbing to such toxins. Because the inner leaflet of the plasma membrane is believed to be less saturated than the outer leaflet (62), increased levels of saturated PA could potentially have large effects. In addition to modulating membrane cholesterol pools, PA may alter intracellular signaling (63), facilitate membrane rearrangements (64, 65), and increase the chaperone activity of heat shock proteins (66). However, these functions have mostly been ascribed to unsaturated PA species, making them less likely as downstream effects of IFN-I.

We show that in addition to inducing increased expression of ACSL1, IFN-I markedly suppresses expression of lipin 1. One of the biological functions of lipin 1 is to catalyze the conversion of PA to DAG (49). The finding that dipalmitoylglycerol (DG 16:0_16:0) was the most abundant DAG species that showed reduced levels in muscle of lipin 1-deficient mice (67) further supports the conclusion that IFN-I uses two complementary strategies to achieve maximal accumulation of fully saturated PA species—induction of ACSL1 and suppression of lipin 1. Together, these results strongly suggest that accumulation of saturated PA is biologically important. Whether IFN-I relies on ACSL1 to generate a PA reservoir for saturated fatty acids and whether this pool has a hitherto unknown function require further studies.

We further provide human relevance by demonstrating that *ACSL1* is induced in PMBCs from individuals with SLE, using a discovery cohort and confirming the results in a replication cohort. Myeloid cells were responsible for the ACSL1 induction in these PBMC preparations. How might the IFN-I-induced changes in glycerophospholipid metabolism be relevant to the pathogenesis of SLE? One possibility is that the changes we describe could lead to impaired lysosomal maturation, which has been proposed to contribute to accumulation of nuclear self-antigens in SLE (68), because downregulation of lipin-1 leads to impaired maturation of autolysosomes (67). Further studies are needed to address this possibility.

Our data demonstrating that loss of ACSL1 causes an increased susceptibility to apoptosis and secondary necrosis, especially under conditions of increased fatty acid load, are consistent with those of Saraswathi and Hasty (69) who showed that the ACSL inhibitor triacsin C results in apoptosis in macrophages exposed to saturated fatty acids. However, others have found reduced cell death in ACSL1-deficient cells stimulated with palmitate and lipopolysaccharide (18, 50) or no differences in cell death in ACSL1-deficient cells (50). The reason for the discrepancies might be due to differences in cell types’ sensitivity to cell death, experimental design, which can markedly impact cell death experiments (70), or to differences in the rates of cell death versus efferocytosis. Strength of our studies include two complementary methods to assess cell death in vitro as well as an in vivo approach (necrosis in lesions of atherosclerosis).

Interestingly, IFN-I-stimulated macrophages rely on fatty acid uptake rather than fatty acid synthesis to meet their fatty acid needs. Thus, de novo 16:0 (palmitate) synthesis is markedly suppressed while total levels of 16:0 are increased in IFN-I-stimulated macrophages (71). Consistently, fatty acid synthase gene expression was suppressed by IFN-I in our study. Therefore, we propose that IFN-I induces ACSL1 to allow channeling of imported saturated fatty acids to a PA reservoir. Without ACSL1 induction, the uptake of saturated fatty acids results in an increased susceptibility to apoptosis and secondary necrosis. Elegant studies have demonstrated that palmitate-induced lipotoxicity associate with accumulation of bi-saturated glycerophospholipid species, rather than of sphingosides or ceramides (72). Interestingly, several of the proteins significantly upregulated by IFN-I only in the ACSL1^M−/−^ macrophages (e.g., CD69 and FN1) were upregulated at the mRNA level by palmitate in that study, consistent with the idea that loss of ACSL1 results in intracellular mislocalization of palmitate and other saturated fatty acids in IFN-I stimulated cells, exacerbating lipotoxicity. Accordingly, the increased cell death in ACSL1-deficient macrophages is most obvious in the presence of increased fatty acid and IFN-I load.

In light of the present findings, increased macrophage death could have contributed to our previous findings of smaller early lesions of atherosclerosis in diabetic mice with myeloid cell ACSL1-deficiency (6), a condition associated with increased IFN stimulation (25). Increased apoptosis might also explain previous findings of suppressed inflammation in the setting of ACSL1-deficiency (6, 15, 16, 73) because rapid efferocytosis of apoptotic cells quells inflammation (74).

In summary, our extensive unbiased characterization of the effects of ACSL1 in myeloid cells shows that IFN-I, and likely other inflammatory mediators (7), use induction of ACSL1 as a strategy to modulate membrane phospholipid composition, rather than to alter macrophage metabolism or inflammatory capacity. In the case of IFN-I, induction of ACSL1 allows an increase in saturated PA species without the risk of apoptosis and secondary necrosis.

## Data availability

If all data are contained within the manuscript and supplemental data; Major Resources Supplement, Phospholipidomics Data Supplement, Proteomics Data Supplement, Metabolomics Data Supplement, Original Data Supplement.

## Supplemental data

This article contains supplemental data.

## Conflict of interest

The authors declare the following financial interests/personal relationships which may be considered as potential competing interests: KEB serves on the Scientific Advisory Board of Esperion Therapeutics.

## Supplemental data

Major Resources Supplement

Phospholipidomics Data Supplement

Proteomics Data Supplement

Metabolomics Data Supplement

Original Data Supplement
