# Supplementary material for: Type I IFN induces long-chain acyl-CoA synthetase 1 to generate a phosphatidic acid reservoir for lipotoxic saturated fatty acids
Source: J Lipid Res. 2024 Dec 14;66(1):100730. doi: 10.1016/j.jlr.2024.100730 (PMC11786746; doi:10.1016/j.jlr.2024.100730)
Supplement: Major Resources Supplement [file mmc1.pdf]

Major resources tables

Antibodies				
	Clone	Final concentration	Product number	Source
MHC class II (I-A/I-E)	M5/114.15.2	1 μg/mL	12-5321-81	Invitrogen
MHC class II	AF6-120.1	1 μg/mL	12-5320-82	eBioscience (Invitrogen)
CD80	16-10A1	1 μg/mL	104707	Biolegend
CD86	GL1	1 μg/mL	105005	Biolegend
CD49D	PS/2	0.1 μg/mL	1520-09	Southern Biotech
CD16/32 Fc Block	2.4G2	1.25 μg/mL	14-016182	eBioscience (Invitrogen)
CD3-PE	17A2	1 μg/mL	555275	BD Bioscience
B220-PerCPCy5.5	RA3-6B2	1 μg/mL	41-0452-82	eBioscience (Invitrogen
Ly6C-PECy7	AL-21	1 μg/mL	560593	BD Bioscience
CD115-APC	AFS98	2 μg/mL	17-1152-82	eBioscience (Invitrogen)
Ly6G-APCCy7	1A8	1 μg/mL	25-9668-82	eBioscience (Invitrogen
CD11b-V605	M1/70	1 μg/mL	101237	Biolegend
Anti-mouse ACSL1 rabbit polyclonal	n/a	1:1,000	4047	Cell Signaling Technology
Anti-lipin 1 antibody				Gift from Dr. Maroun Bou Kahlil (University of Ottawa)
Anti-lipin 2 antibody				Gift from Dr. Brian Finck (Washington University)
Anti-mouse Mac-2 rat IgG2a	M3/38	1 µg/mL	CL8942AP	Cedarlane
Anti-mouse β-actin monoclonal antibody	AC-15	1:10,000	A5441	Sigma-Aldrich
HRP-conjugated secondary antibody-mouse		1:10,000	7076	Cell Signaling Technology
HRP-conjugated secondary antibody-rabbit		1:10,000	7074	Cell Signaling Technology

Primers (mouse)		
Gene	Forward primer	Reverse primer
Acsl1	CTACTACGACGATGTCAGAACCA	GGAAATCCACTCATAGGGCTGG
Ccl2	TTAAAAACCTGGATCGGAACCAA	GCATTAGCTTCAGATTTACGGGT
Ifnar	AGCCACCGAGAGTCAATGG	GCTCTGACACGAAACTGTGTTT
Irf7	TCCAGTTGATCCGCATAAGGT	CTTCCCTATTTTCCGTGGCTG
Lipn1	CTCCGCTCCCGAGAGAAAG	TCATGTGCAAATCCACGGACT
Lpin2	GAAGTGGCGGCTCTCTATTTC	AGAGGGTTACATCAGGCAAGT
Isg15	GGTGTCCGTGACTAACTCCAT	TGGAAAGGGTAAGACCGTCCT
Rn18s	CATTAAATCAGTTATGGTTCCTTTGG	CCCGTCGGCATGTATTAGCT
**Primers (human)**		
Gene	Forward primer	Reverse primer
ACSL1	AACAGACGGAAGCCCAAGC	TCGGTGAGTGACCATTGCTC
IRF7	CCCACGCTATACCATCTACCT	GATGTCGTCATAGAGGCTGTTG
ISG15	GAGAGGCAGCGAACTCATCT	AGCATCTTCACCGTCAGGTC
RN18S	Hs9999901_s1	

Animals				
Species	Vendor or Source	Background Strain	Sex	Persistent ID / URL
Mouse	Jackson Laboratory	C57BL/6J	F, M	000664

Major resources tables

Mouse	Our breeding colony *Acsl1*$^{fl/fl}$ mice originally from R. Coleman	C57BL/6J, *Acsl1*$^{fl/fl}$ *Lyz2-Cre*$^{Tg/Tg}$ and *Acsl1*$^{wt/wt}$ *Lyz2-Cre*$^{Tg/Tg}$	F, M	
Mouse	Jackson Laboratory	C57BL/6J, *Ldlr*$^{-/-}$	F, M	002207
Mouse	Originally from J. Sprent	C57BL/6J, *Ifnar*$^{-/-}$	F, M	
Mouse	Originally from M.S. Diamond	C57BL/6J, *Irf3/7*$^{-/-}$	F, M	

Reagents		
Description	Source / Repository	Persistent ID / URL
Universal IFN-I	PBL	11200
Resiquimod (R848)	Tocris	4536
IL-18 ELISA	Invitrogen	88-50618-88
S100A8/A9 ELISA	RnD	DY8596
Cholesterol E Assay	Wako Diagnostics	999-02601
Amplex Red cholesterol assay kit	ThermoFisher	A12216
Stanbio™ Triglyceride kit	Fisher Scientific	2100430
DNA isolation and purification kit	QIAGEN	158043
Standard laboratory diet (chow)	Purina Mills	5053
Viability dye e450 for flow cytometry	eBioscience	65-0863-14
Clarity Western ECL Substrate	Bio-Rad	1705060
Membrane fluidity kit	Abcam	ab189819
Pristane (tetramethylpentadecane)	Sigma-Aldrich	P2870
